# Current challenges and best-practice protocols for microbiome analysis

**DOI:** 10.1093/bib/bbz155

**Published:** 2019-12-18

**Authors:** Richa Bharti, Dominik G Grimm

**Affiliations:** Weihenstephan-Triesdorf University of Applied Sciences and Technical University of Munich, TUM Campus Straubing for Biotechnology and Sustainability, Straubing, Germany

**Keywords:** microbiome, amplicon sequencing, 16S rRNA sequencing, metagenomics, assembly, functional and taxonomic classification

## Abstract

Analyzing the microbiome of diverse species and environments using next-generation sequencing techniques has significantly enhanced our understanding on metabolic, physiological and ecological roles of environmental microorganisms. However, the analysis of the microbiome is affected by experimental conditions (e.g. sequencing errors and genomic repeats) and computationally intensive and cumbersome downstream analysis (e.g. quality control, assembly, binning and statistical analyses). Moreover, the introduction of new sequencing technologies and protocols led to a flood of new methodologies, which also have an immediate effect on the results of the analyses. The aim of this work is to review the most important workflows for 16S rRNA sequencing and shotgun and long-read metagenomics, as well as to provide best-practice protocols on experimental design, sample processing, sequencing, assembly, binning, annotation and visualization. To simplify and standardize the computational analysis, we provide a set of best-practice workflows for 16S rRNA and metagenomic sequencing data (available at https://github.com/grimmlab/MicrobiomeBestPracticeReview).

## Introduction

The recent advances in high-throughput sequencing helped to unfold the most abundant living material, the ‘microbiome’, and its associations to different environments. The microbiome exists as an essential component of diverse habitats including air, soil, water and the gut of simple and complex organisms [1, 2]. It plays crucial roles in metabolic processes of both abiotic and biotic systems including mineral recycling and breakdown, nitrogen fixation, as well as modulation of host immune responses and production of vitamins and secondary metabolites [3, 4]. Eventually, recognition of the diverse roles of microbes in numerous biotic and abiotic systems has expanded the scope of microbiology beyond laboratory-grown cultures. It might have helped to redefine the previously conceptualized idea of ‘holobiont’ that incorporates specific host–microbe symbiotic associations into a more generalized and inclusive ‘hologenome’ [5–7]. Hologenome describes the genetic totality of host genes and symbiotic/mutualistic microbial genes that get affected simultaneously under environmental stress [8]. Studies on understanding the roles of the hologenome got boosted with advancements in next-generation sequencing (NGS) that helped to precisely identify microbial species and associated metabolic pathways [5, 9, 10]. In the past 15 years, the Human Microbiome Project and Earth Microbiome Project together with NGS immensely improved the areas of novel genome predictions, genetic associations, pathogen identifications and clinical diagnostics [11, 12]. Nonetheless, there have been concerns about the reproducibility of published microbial sequencing data that consist of large amounts of unknown sequences, also referred to as ‘dark matter’ [11, 13]. Erroneous sample handling, variation in sampling size, choice of DNA extraction methods, as well as computational analyses (e.g. quality filtering tools and assemblers) might lead to inconsistent results. In addition, the lack of standardization of laboratory and computational protocols introduces various biases, which then might lead to non-comparable results.

This review discusses both the experimental and computational challenges in acquisition and analysis of 16S rRNA and metagenomics data while focusing on the advantages, limitations and best practices for data handling and analysis. The article begins with a review of gene amplicon and metagenomic sequencing methods and their experimental challenges followed with a best-practice bioinformatics analysis workflow for standardizing the analysis, as well as for achieving robustness and reproducibility.

## NGS-based microbial genotyping

**Figure 1:**
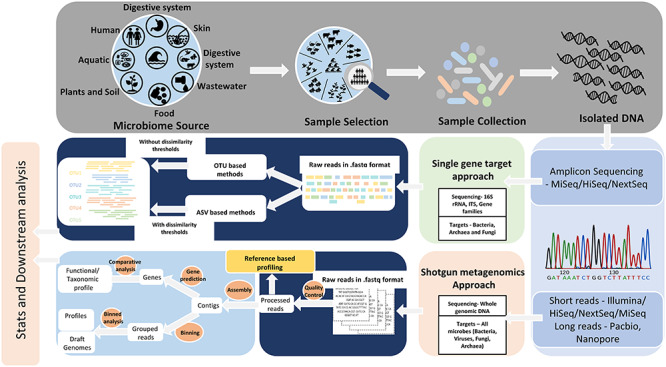
An illustration of targeted amplicon and metagenomic sequencing approaches. A schematic overview demonstrating diverse sample types along with commonly utilized sequencing platforms, as well as systematic and stepwise data processing steps.

The two most commonly used methodologies for microbial identification and genotyping are based on gene amplicon/marker genes (e.g. 16S rRNA) and shotgun metagenomics (Figure 1).

### Gene amplicon sequencing

Over the past 25 years, gene amplicon sequencing has been the primary technique to study phylogeny and taxonomy of complex microbiomes that were earlier considered challenging to characterize [14]. For bacteria, archaea, fungi and mycobacteria, several specific marker/target genes are identified that are extensively used for amplicon sequencing. Most marker genes are functionally conserved across phylogenetic distances and thus also serve as a molecular clock for studying evolutionary transitions and changes. The most commonly used target gene for bacterial identification is 16S rRNA (or 16S rDNA), which is the gold standard in microbial typing [15, 16]. The 16S rRNA gene encodes prokaryotic small 30S subunit of the 70S ribosomal complex in most bacteria and archaea. Interestingly, the prokaryotic 16S rRNA gene is distinct from its eukaryotic homologue, the 18S rRNA gene that encodes the small eukaryotic ribosomal subunit (40S). The highly conserved 16S rRNA gene implies its crucial role in cellular function and survival and thus forms the basis of obtaining precise genomic classification of known and unknown microbial taxa. Additionally, it is easier to sequence 16S rRNA genes even for exceedingly large sample sizes, due to its relatively short size (~1542 bp). The gene sequence consists of highly conserved primer binding sites along with nine variable regions (V1–V9). Most of the 16S rRNA-based genotyping protocols use V5–V6, V3–V4, or V4 hypervariable regions to identify and catalogue microbial profiles [17, 18]. Alternatively, the V3 region is a better choice for community profiling of *Archaea* by polymerase chain reaction (PCR)–denaturing gradient gel electrophoresis. Other variable regions, including V1–V2 and V3–V4, have been utilized for genotyping archaeal species in complex microbial communities [19]. Unlike bacteria, identification of gene targets in pathologically important yeast and fungi is still not well determined. The fungal rDNA is composed of coding and noncoding spacer regions [20, 21]. The coding region consists of 18S, 5.8S and 28S units along with several noncoding regions consisting mainly of internal transcribed spacers (ITSs) and intergenic sequences. ITS variable regions have been the most commonly used gene target for fungal genotyping. However, uneven lengths of these ITSs induce errors and biases, such as preferential amplification and sequencing, often leading to an incorrect estimation of abundance [21].

Nonetheless, 16S rRNA-based NGS has been successfully used in characterizing microbial communities associated with various milieus including soil, water sources and the human gut (Figure 1). More recently, 16S rRNA-based NGS analysis has helped to identify changes in microbial community structures along with its associated alterations in community functions. It helped remarkably in the estimation of soil and water contamination, as well as to gain a deeper understanding of several gut-associated diseases, including Crohn’s disease, ulcerative colitis, diabetes and gastrointestinal cancers [22–27].

### Metagenomics

Metagenomics refers to direct genetic analysis of genomes obtained from different environments [28]. The term metagenomics is often used inaccurately in conjunction with 16S rRNA gene sequencing. While 16S rRNA sequencing utilizes a marker gene approach and does not target the whole genome, metagenomics on the contrary is a culture-independent genomic analysis of microbes taken directly from the environment using a genome-wide shotgun sequencing approach [29, 30]. Metagenomics comprehensively catalogues all microorganisms present (unculturable and culturable, known and unknown) in complex environmental samples (Figure 1). In contrast to the unimodal phylogenetic analysis based on the diversity of a single gene, for instance, the 16S rRNA gene, metagenomics systemizes multimodal genetic composition of microbial communities and hence provides a better taxonomic resolution and genomic information [31, 32]. Metagenomics helps in associating function to phylogeny besides creating evolutionary profiles of the microbial community structure. Importantly, it also helps to identify viruses that are otherwise hard to detect through a single-gene targeting approach, due to its high genetic diversity and its inability to discern common genetic links [33]. In the past few years, modern NGS has slowly replaced classical Sanger sequencing as a preferred tool for metagenomics shotgun sequencing. Both 454/Roche and Illumina/Solexa systems were extensively used for analyzing metagenomic samples from a multitude of environments [34].

**Figure 2:**
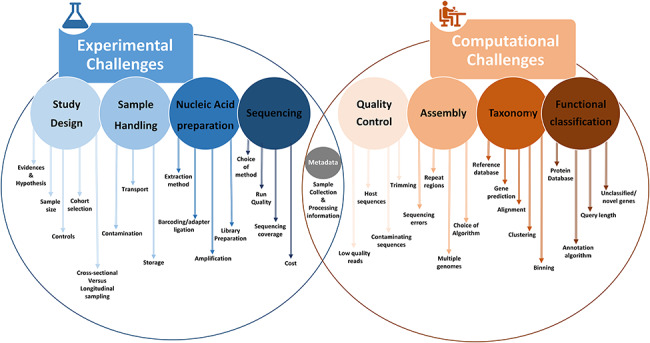
A schematic overview outlining various experimental and computational challenges associated with 16S rRNA-based and shotgun metagenomic sequencing.

Despite the recent advancements in sequencing technologies and computational analysis tools, many factors might lead to biases and errors. These errors and biases could be broadly classified into experimental and computational challenges. Figure 2 shows a general overview of common experimental and computational challenges that are discussed in detail in the following sections.

## Experimental challenges and solutions

### Study design/experimental design

A good study design helps in limiting erroneous and obscure trends often observed in several microbiome-based studies. In general, any hypothesis should primarily be supported by meticulous literature driven evidence and preliminary testing using small-scale/pilot studies to avoid uncertainty in biological signals, trials and failures. A rationalized study design will certainly help to improve data processing and to eliminate confounding effects [35]:

- (i) Number of samples: Selecting a significant sample size remains a key step, especially when the final outcomes are used for clinical settings and interpretations. It has been reported that the microbial load varies between biological replicates existing under similar conditions [36]. This variability between similar samples makes it challenging to identify weak biological signals, especially when the effective size is unknown or small. In most cases, results with small sample sizes do not precisely represent general population-based outcomes. Importantly, sample sizes should always be kept fixed and should not be altered during the study [37]. Hence, choosing appropriate sample sizes based on statistical principles can certainly help to avoid biases and spurious interpretations.
- (ii) Controls: Controls are needed to identify whether a signal is real and not just a stochastic or spurious result. An appropriately controlled experiment consists of two or more scenarios: one producing observations without interferences, while the others remain targeted manipulations [38, 39]. Unfortunately, it is still a difficult task to obtain proper controls in many cases, especially in clinical trials where the microbial composition gets affected by age, gender, ethnicity, diet, genotype and several other lifestyle factors. In animal studies, additional factors, such as animal strains, facilities, housing conditions, handling and breeding, could also affect the microbial profile [40]. Several studies have shown that co-housed animals could act as hidden confounding factors due to coprophagy [41, 42]. Thus, it is essential to replicate a co-housing study making sure not to co-house animals of different genotypes, which might have different phenotypic appearances. On the other hand, it has also been found that genetically identical mice in different facilities exhibit different bacterial profiles [42]. Nonetheless, one should try to control and document as many factors as possible to create a detailed metadata file (Supplementary Table S1). These factors could later be used in statistical downstream analyses to account for confounding factors [43, 44].
- (iii) Cross-sectional and longitudinal studies: A cross-sectional study incorporates comparative analyses of two groups, e.g. healthy versus disease or treatment versus placebo. These studies are less complex to design and perform and do not essentially require long follow-ups. However, a significant drawback of such studies is that observed differences are not directly attributed to a single effect/treatment and could be due to various additive or multiplicative effects [45]. It is well known that a microbiome could be altered based on many environmental factors that include lifestyle and diet. Hence, from a statistical perspective, it is better to perform longitudinal studies, where the same sample is studied under different controlled conditions [46]. However, it is equally important to cautiously plan identical sample collection times for each replicate to avoid biases. Despite the advantages associated with longitudinal studies, only a few reliable methods are available for downstream analyses [47].
- (iv) Metadata: Metadata are an information catalogue containing details of all the samples used in an experiment. Generation of metadata is one of the most critical steps before any downstream analysis could be performed. Apart from serving as a sample reference sheet, it also helps to avoid false interpretation of results and highlights the effective size of individual factors. The use of metadata is needed in several modern statistical comparison tools [48]. An example metadata sheet is provided as Supplementary Table S1 for reference.

### Sample collection and handling

Handling of environmental samples after collection is a crucial aspect in nucleic acid-based sequencing methods for comparing the composition and diversity of microbial communities. In fact, sample collection could be a significant confounding factor that might affect the results and interpretations of a study [49]. The most common problem is variability in the amount of microbial DNA present in different environmental samples. For example, skin samples contain comparatively less microbial biomass than gut samples, and hence collecting enough samples remains a crucial factor for the final sequencing outcomes. In the following we list some parameters which should be considered during sample collection and handling:

- (i) *Contamination*: Maintaining a proper sample environment during sample collection is important, since changes in temperature, humidity, or other factors could alter or contaminate samples [49]. Additionally, the proximity of different samples could lead to cross-contamination, which might later generate spurious results. Furthermore, minimizing the time of sample collection and using aseptic laboratory resources, including gloves, masks and head covers, help to reduce contamination [50].
- (ii) *Transportation*: Transit conditions and duration can influence the quality and quantity of extracted nucleic acids. The microbial composition is unstable from the point of sample collection, and thus immediate freezing is considered as a must. It has been shown that the interim period between sample collection and storage can lead to several issues in later phases of the analysis [51]. Thus, it is crucial to maintain constant storage conditions during transportation for all samples to avoid inconsistent freeze–thaw cycles. Additionally, several chemical preservation methods are widely accepted for sample collection from remote locations [52, 53].
- *Storage and safety*: Several studies have assessed the effect of storage conditions on compositional changes in microbial samples. Comparing 16S rRNA profiles, it has been shown that short-term (14 days) storage temperature has an insignificant effect on the microbiome structure and diversity in samples [54]. Another study on human fecal microbiota showed that rapid refrigeration at −80°C conserves microbiota diversity that is significantly altered by dry storage at 4°C [53]. Hence, it is equally important to maintain consistent storage conditions for obtaining optimal nucleic acid yields before sequencing.

### Nucleic acid extraction

The choice of DNA/RNA isolation methods could cause biases during sequencing, which in turn affects downstream analysis. Importantly, the extraction method should effectively capture all types of microbes. For example, DNA isolation from gram-positive bacteria is harder, due to their thick peptidoglycan cell walls [55]. There are two major extraction methodologies: (i) mechanical lysis/bead beating and (ii) chemical lysis [56]. Bead-beating methods are considered to produce superior yields if done optimally. Thus, for complex bacterial samples, a ‘bead-beating’ step could be performed before standard nucleic acid extraction. However, vigorous bead beating should be avoided since it can shear nucleic acids and eventually affect library preparation steps later.

### Nucleic acid preparation

For single marker/target gene NGS approaches, amplification using barcode primer pairs, purification, and preparation of purified DNA libraries are done before sequencing. Illumina MiSeq provides a limited output (15 Gb) and is mainly used for amplicon sequencing as it provides longer reads (2× 300 bp) with a much lower sequencing cost compared to other high-throughput sequencers [57]. Interestingly, Illumina also offers shotgun sequencing which generates short reads up to 1.5 Tb per run. Multiple DNA isolation methodologies are available that differ based on fragmentation methods and efficiently generate sequencing libraries. Widely used DNA isolation kits for the Illumina platform include Nextera DNA Flex, Nextera XT and TruSeq DNA PCR-Free [58]. Nextera DNA Flex supports both large and small genome sizes with input DNA amounts of 100–500 ng and 1–500 ng, respectively. It utilizes bead-linked transposomes that simultaneously generate consistent fragment sizes and tag the input DNA. Up to 96 multiplexed metagenomic samples can be sequenced using unique dual indexing during library preparation. Another popular kit, Nextera XT, utilizes an engineered enzyme-mediated fragmentation methodology and requires as little as 1 ng of input DNA samples. Using this method, up to 384 uniquely indexed samples can be pooled and sequenced together. On the other hand, TruSeq DNA PCR-Free as the name suggests is a PCR-free workflow and utilizes mechanical DNA fragmentation and adapter ligation. This method also requires little amounts of input DNA (~1 ng).

Unfortunately, short-read-based NGS techniques have limited applications in analyzing polyploid genomes due to pure applicability of their algorithm to metagenomics data. In this context, third-generation sequencing platforms like Pacific Biosciences RS II/Sequel and Oxford Nanopore MinION sequencing technologies prove to be more efficient due to longer read sizes, species-level resolution and absence of DNA amplification-based biases [59, 60]. Pacific Biosciences RS II/Sequel has improved extraction procedures that incorporate enzymatic lysis of DNA with a cocktail of enzymes that results in the extraction of longer DNA fragments. Additionally, in comparison to PacBio RS II, the PacBio Sequel has raised DNA output from ~0.5–1 Gb to ~5–10 Gb [61]. Nevertheless, conventional glitches like collection, preservation and transfer can still retrograde sample quality and have caused a multitude of problems in exploring clinical samples and samples from extreme environments. Subsequently, another third-generation sequencing technology, the Oxford Nanopore MinION, reliably addresses these issues [62, 63]. Launched in 2014, it is portable (size of a USB stick) and provides the agility to sequence samples from extreme conditions. Nonetheless, read lengths produced by the MinION nanopore sequencer rely mostly upon input fragment lengths that again require meticulous extraction and purification procedures.

## Sequencing and computational challenges

Recent developments in sequencing technologies have resulted in an exponential increase in new methods, algorithms and computational tools for functional annotations and analyses [64]. However, several computational challenges still exist due to the complexity of the underlying biological data, lack of proper metadata information and scarcity of standard data formats and computational resources for high-volume data (Figure 2) [65, 66]. Since most of the biological interpretation of sequencing data relies on these tools, proper benchmarking, open-source availability, simplification of the installation process and a proper user interface should help to ensure reproducibility and interpretability of the results. This is important, since using different tools for similar analyses often results in different and non-comparable results, interpretations and biases. Hence, it is crucial for research projects that are heavily dependent on bioinformatics tools to access and utilize these tools conscientiously. There are various computational tools for 16S rRNA sequencing data [67, 68], as well as for short-read metagenomics data (e.g. *Critical Assessment of Metagenomic Interpretation* (CAMI)) [69, 70]. In the following sections, we provide an overview of current challenges in amplicon and metagenomic sequencing analysis followed by a best-practice workflow on how to optimally conduct such analyses.

### Challenges for amplicon sequencing analysis

One of the main difficulties for gene marker-based analysis is to distinguish sequencing errors from real nucleotides. For this purpose, two major tool categories exist: (i) operational taxonomic unit (OTU)-based (QIIME and Mothur) [159–160] and (ii) amplicon sequence variant (ASV)-based (DADA2, Deblur, MED,and UNOISE) [158, 177–179] tools (Figure 1). OTU-based methods resolve sequencing errors by clustering the reads based on a predefined identity threshold (commonly 97%) into OTUs [71]. On the other hand, ASV-based tools utilize a denoising approach on biological sequences before the introduction of amplification and sequencing errors [72]. Several comparative studies between these two methods have suggested that OTUs provide lower taxonomic resolution as compared to ASVs and a choice between these two can broadly impact alpha diversity estimations [73, 74–78]. In this review, we describe a stepwise systematic workflow for 16S rRNA using OTU- and ASV-based methods, in the forthcoming section.

### Challenges of metagenomic sequencing analysis

A rapidly growing number of tools and algorithms available for metagenomic analyses have made the choice of the most appropriate methods highly challenging. Major steps involved in typical metagenomics data analyses are assembly and binning, followed by taxonomic and functional profiling (Figure 1). In the following subsections, these steps are comprehensively discussed followed by a description of a systematic workflow containing optimal tools and algorithms.

#### Quality control

Quality control is an essential prerequisite that involves quality trimming and contamination removal from raw reads. While quality trimming filters raw reads for low-quality and adapter sequences, contamination removal detects and efficiently removes host-associated sequence contaminations from reads. Both steps are crucial for producing an optimal assembly. Trimmomatic, *sickle*, BBTools and DeconSeq are widely used tools that utilize bowtie and BWA for quality trimming and contamination removal [74–77]. Next, a variety of read lengths generated from an environmental sample are processed through either *short-read* or *long-read metagenomic analyses* depending on the study design.

**Figure 3:**
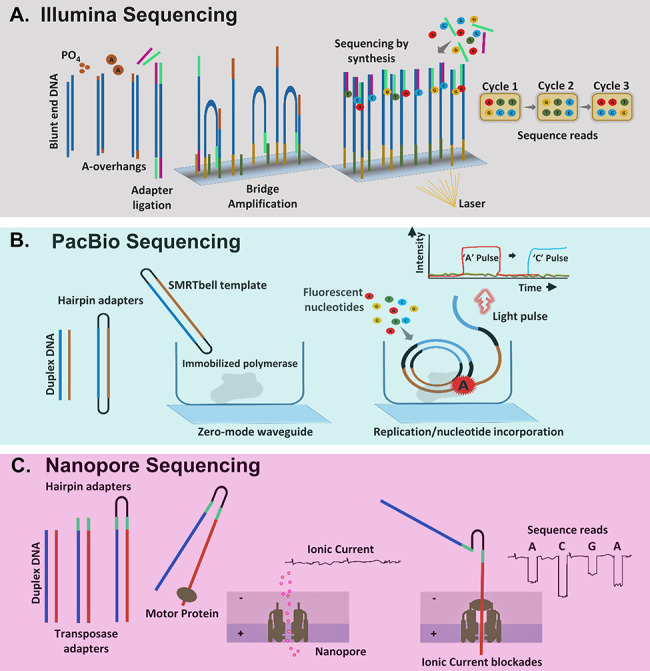
Major short-read and long-read sequencing technologies. (**A**) Illumina sequencing involves initial trimming, adenylation of the blunt ends and ligation of specific adapters to DNA molecules. Following this library, fragments are amplified *in situ* on flow cell surfaces through bridge amplification and produce sequencing clusters. Finally, reversible dye terminator sequencing step is implemented where single-nucleotide addition reactions and presence of blocking group at the 3′-OH (of the ribose moiety) help to identify sequencing clusters through a reporter fluorescent signal. (**B**) PacBio sequencing involves a circular consensus sequencing (CCS) SMRTbell technique. Herein, ligation of hairpin adapters to each end of a duplex DNA molecule forms a closed loop, which is sequenced in a zero-mode waveguide (ZMW), fluorescence-based readout of nucleotide incorporation. Each strand in the duplex DNA is sequenced together in multiple passes, and the consensus sequences from both strands are incorporated. (**C**) Nanopore sequencing involves ligation of hairpin adapters at one end of duplex DNA molecule before initiating nanopore sequencing of the linked original DNA strands. The blockades in ionic current through the nanopore are optimally quantified as DNA base sequences.

### Challenges in short-read metagenomics

The primary advantage of short-read sequencing is its ability to generate billions of reads in a massively parallel manner within a single run. The Illumina sequencing platform is a short-read technology that produces high read counts at comparatively lower costs. In Illumina sequencing, an adapter-ligated DNA library is captured using surface-bound complementary oligonucleotides and later amplified into distinct, clonal clusters by bridge amplification (Figure 3A). Sequencing is done in multiple cycles by imaging a fluorescently labeled reversible terminator after each dNTP addition, which is then cleaved to allow incorporation of the next base. This process minimizes errors due to a base-by-base sequencing protocol that enables accurate data acquisition. In the following, a stepwise metagenomics processing workflow is discussed along with suggestions of optimal tools and algorithms.

#### Assembly

Significant challenges in analyzing complex environmental samples comprising multiple genomes (bacteria, fungi, viruses, archaea) include sequencing errors, presence of intergenomic and intragenomic repeats and uneven sequencing coverage [78, 79]. The assembly step fairly subjugates these issues by stitching reads into longer fragments, referred to as contigs, followed by reconstructing the individual genes and species. Reads can be either paired-end reads, mate pairs, or single-end reads based on the choice of adapter ligation [80]. Further, the complexities and challenges for metagenomics assembly are elevated by an uneven abundance of multiple genomes in samples originating from the same conditions. Hence, the choice of the assembly algorithm remains critical for optimal downstream analysis. For a typical metagenomics assembly, commonly used assemblers include MegaHit, metaSPAdes, RayMeta, MetaVelvet, IDBA-UD, SOAPdenovo2 and Omega [81–87].

Interestingly, most of these assembly algorithms were initially developed for single-genome assemblies but have been extended for a much broader usage. Apparently, the choice of the right algorithm for a given dataset has become difficult due to numerous comparative reports on these different assemblers [88, 89]. Several efforts have been made to improve assembly statistics and identification of dedicated metagenomic assemblers. The four most widely used assemblers are MegaHit, metaSPAdes, RayMeta and IDBA-UD. All four algorithms are open-source metagenome assemblers based on *De Bruijn graphs* [90]. A major advantage of *De Bruijn graphs* is that assembled reads contain fewer errors and errors can be easily corrected prior to assembly. On the other hand, the IDBA assembler iterates through increasing *k*-mer sizes, trimming the graph and integrating bubbles/loops along the way*.* It utilizes several depth-relative *k*-mer thresholds for eliminating erroneous *k*-mers in both low-depth and high-depth regions*.* Similarly, while RayMeta is a single *k*-mer assembler, both metaSPAdes and MegaHit iteratively analyze *k*-mer lengths to find the optimal value. While metaSPAdes incorporates read coverage during assembly, MegaHit has a comparatively rapid and memory-efficient implementation. Both assemblers are preferred for complex microbiome profiling tasks [91].

#### Gene prediction

Several methods for predicting genes from metagenomic DNA fragments are available that are widely used. These prediction algorithms are broadly categorized into homology-based, model-based and machine learning-based methods [92]. Currently, gene prediction tools such as GeneMarkS, Glimmer3 and Prodigal exhibit significantly high accuracy (>97%) in detecting validated protein-coding ORFs [93–95]. Nevertheless, their accuracy in identifying the ORF-start sites could still be improved (~90%) [93]. This is mainly because genes escaping the detection are the genes with sequence patterns that do not match any species-specific model [96]. Additionally, another critical accuracy measure, the false-positive rate (FPR), requires a rather difficult assessment for ruling out wrongly predicted genes. A robust way of detecting false positives based on a two-factor assessment has been recently proposed that involves (i) identifying large overlaps with known genes located on the opposite strand and (ii) comparing with genes predicted in random sequences [97].

#### Contig binning

In order to reconstruct genomes using heterogeneous sequencing data, contig grouping based on an individual genome of origin or metagenomics binning is done. Traditionally, binning is performed by aligning contigs against reference datasets, but recently more efforts were directed toward unsupervised clustering [98]. Hence, binning algorithms can be further categorized as either taxonomy-dependent or supervised binning that utilizes taxonomic information from a reference database or unsupervised binning where sequence clustering is performed using statistical properties and/or contig coverage [99]. In supervised or taxonomy-dependent binning, contig classification reduces the search space, and thus slower alignment or phylogenetic methods can be executed. In this context, the widely used tool Taxator-tk utilizes Basic Local Alignment Search Tool (BLAST) and PhyloSift for identifying similarities to marker genes (such as 16S rRNA) using the Hidden Markov model profiles [100]. Similarly, other tools including HMMER and PhyloPythiaS(þ) assign reads to bins by utilizing an support vector machine model trained on a reference database [101, 102].

On the other hand, unsupervised binning mostly relies on sequence features without a priori information on genome sets present in a sample. For instance, MetaCluster bins reads by a dual grouping algorithm, where it first groups reads using long unique *k*-mers (*k* > 36) followed by merging groups based on similar tetranucleotide or pentanucleotide distributions [103]. In the next round, 16 mer frequencies are utilized to bin contigs from low-abundance species. Apart from these, three other metagenomic contig binning algorithms include MaxBin, CONCOCT and MetaBAT. MaxBin considers nucleotide composition and contig abundance information for binning through an expectation–maximization (EM) algorithm that precisely clusters metagenomic contigs into bins consisting of contigs from a single species [104]. On the contrary, CONCOCT uses Gaussian mixture models for clustering contigs by combining both tetranucleotide frequencies and differential abundances covering multiple samples for binning [105]. Thus, it amalgamates information from both sequence composition and coverage, across multiple environmental samples. Similarly, MetaBAT utilizes pairwise collation of contigs by calculating probabilistic distances based on tetranucleotide frequencies. Binning of contigs is then done by a *k*-medoid clustering algorithm and modeled on interspecies and intraspecies distances in the sequenced genomes [106].

#### Taxonomic classification

For identifying the taxon of each sequence, most metagenomic classification tools match sequences (reads or contigs) to known microbial genome databases. Due to the rapidly increasing size of sequencing datasets, the canonical BLAST-based alignment of sequences to GenBank has become impracticable [89]. Several metagenomics classifiers are available that provide faster analyses at the expense of sensitivity. These classifiers utilize a variety of approaches including simple read alignments, *k*-mer mapping in whole genome sequencing reads, alignment of marker genes only, or generating translated protein sequences and their alignment to protein databases [107]. Perhaps, marker gene approaches allow faster taxonomic assignments, due to their comparatively smaller sequencing data that can be aligned against databases incorporating full genomes of maximum species. Eventually, several fast aligners like Bowtie2 [108] and HMMER [109] are utilized by several other tools, such as MetaPhlAn [110], Phylosift [111] and mOTU [112]. Another tool, *GOTTCHA*, employs 24 unique base-pair fragments indexed with BWA (Burrows–Wheeler alignment)–mem (maximal exact matches) that helps to generate either a presence-/absence-based binary classification or complete taxonomic profiles [113].

On the contrary, for metagenomics data, Kraken was the first algorithm that provided fast identification of all reads and relied on exact *k*-mer matches between lowest-common ancestor (LCA) of every taxon [114]. Another tool CLARK utilizes a modified approach of keeping only species or genus-level *k*-mers and discarding the rest of the *k*-mers that map to higher taxonomic classifications [115]. Apart from these, few other tools, such as Centrifuge [116], MEGAN6 [117], taxator-tk [100], DUDes [118] and Taxonomer [119], also exist for taxonomic classifications of metagenomics data. While Centrifuge is a compact, metagenomics classifier that utilizes Burrows–Wheeler transform with FM index for indexing a genome database, both MEGAN6 and taxator-tk extensively use the outputs of local sequencing algorithms including BLAST [120], DIAMOND [121], or LAST [122]. On the other hand, DUDes is an unambiguous classifier that utilizes the output of read aligners such as BWA–mem for interpreting taxonomic abundances in samples [118]. Similarly, Taxonomer is a rapid and ultrasensitive classifier that first bins reads into broad ranges, followed by their separation into species-level messenger RNA (mRNA) transcript profiles [119].

#### Functional classification

Functional classification of metagenomics data is vital for investigating the functional and metabolic roles of microbiome member species, as well as their variations under different conditions/treatments. Overall, tools for functional classification share common features with tools used for whole genome analyses **(**Figure 3**)**. These tools and approaches can be classified into four major categories, *viz.*, homology-based, motif- or pattern-based, context-based and other functional predictions:

(i) Homology-based tools: This is one of the earliest approaches for which predicted protein sequences are matched to reference protein sequences, such as *NCBI RefSeq* [123], *UniProt* [124] and *SMART* [125]. Both IMG/M [126] and MG-RAST [127] servers allow query matching with other databases, including clusters of orthologous groups (COGs) [128], *Pfam* [129] and *TIGRFAM* [130]. Significant disadvantages of this approach are long computation times and high error rates (~15%), due to database propagation.
(ii) Motif- or pattern-based tools: This approach is suitable for short reads and complex samples that could not be matched using homology-based approaches. Databases like *PROSITE* [131], *PRINTS* [132], or *InterPro* are utilized to screen common motifs in metagenomic sequences. IMG/M does most of the motif-/pattern-based annotations but with low statistical significance and high false-positive rates.
(iii) Context-based tools: Novel metagenomic sequences that do not share any homology nor pattern or motif from the two previous approaches are processed using a context-based annotation. This approach mainly utilizes genomic neighborhoods for screening metagenomic sequences. Both IMG/M and SmashCommunity are commonly used context-based mapping algorithms [133].
(iv) Other functional predictions: Putative annotations of novel metagenomic sequences are usually performed using specific tools for predicting functional properties, such as carbohydrate-active enzymes (CAZy), protein localizations (PSORT, CELLO), lipoproteins (DOLLOP, Lipo, SignalP), insertion sequences (ISsaga) and virulence factors (VFDB, MvirDB) [134–137].

### Metagenomics challenges with long reads

Long reads are crucial for deciphering genomic regions that remain inaccessible to short-read sequencing, due to the presence of repeated sequences. Apparently, it also helps in sequencing entire RNA transcripts and provides precise information on the existence of specific isoforms [138]. Although second-generation sequencing technologies, such as Roche 454 and Ion Torrent, generate effectively longer read lengths (~700–1000 bp), they are usually not preferred, because of high sequencing costs and the generation of homopolymers. Illumina platforms provide higher accuracies and are more cost-effective; however, they only provide limited read length (~2× 300 bp). At present, both Pacific Biosciences single-molecule real-time (SMRT) and Oxford Nanopore Technologies sequencing platforms are preferred due to their longer read sizes of 15–100 and ~1000 kilobases, respectively [60, 139].

#### PacBio sequencing

PacBio is a third-generation sequencing platform that utilizes sequencing by synthesis workflow like Illumina, except that it is a single-molecule real-time (SMRT) sequencing technology **(**Figure 3**B****)**. The PacBio-produced SMRT technology employs (a) an SMRT Cell in the form of zero-mode waveguide that allows observation of individual fluorophores and maintains a high signal-to-noise ratio, (b) fast and accurate synthesis reaction by phospho-linked nucleotides and (c) real-time, continuous light pulse-based signal detection. This results in an accurate and very high-throughput DNA sequencing at a low cost. Another huge advantage of PacBio is its ability to produce much longer reads ranging between 10 and 50 kbp with an average read accuracy of ~85% [140]. Currently, in comparison to PacBio RS II, the new PacBio Sequel System shows a significant increase in read lengths (~0.5–10 Gbp). The recent incorporation of a hybrid error correction method (PBcR—PacBio corrected reads) led to an improved read accuracy from 80% to 99.9% [61, 141]. Additionally, the hierarchical genome assembly process (HGAP) has ended the requirement of high-quality reads to reconstruct the genome [142]. In this method, the longest read among the datasets is selected as a ‘seed’, and all other reads are mapped against it. Later, a preassembly is done to convert the seed reads into precise preassembled reads that can be used for a genome assembly. Finally, a refinement of the assembly is done by using the initial reads which generate a consensus read sequence. Although the assembly of SMRT reads with HGAP produces a precise assembly of high-coverage regions, it subsequently fails to reconstruct low-coverage regions from complex communities. More recently, a postprocessing step using BIGMAC (breaking inaccurate genomes and merging assembled contigs) was introduced, where both contigs and original reads were simultaneously used for an improving *de novo* assembly [143]. Overall, the PacBio platform is highly advantageous for studying *de novo* genomes, transcriptomes and direct epigenetic characterizations.

Also, for complex microbial populations, PacBio offers full-length gene profiling of ITS or 16S rRNA regions. It can also effectively perform full-length transcriptome profiling of eukaryotic samples in a row at once [144, 145]. Interestingly, a comparative study combining Illumina short reads and PacBio long reads from Marine sponges showed that the hybrid approach and phylotype-specific bins helped to improve the assembly quality and statistics and could be used as a complementary technique for variant calling in SMRT [146]. Moreover, low-depth SMRT data can also precisely reconstruct taxonomic profiles of complex communities and can also generate highly accurate closed genomes, as demonstrated in a study on human skin metagenomes [147]. Also, a few other recent reports showed that PacBio shotgun metagenomics could precisely identify dominant species from low diversity microbial communities and can also effectively recover rare genomes as compared to other short-read platforms [148, 149].

#### MinION nanopore sequencing

Several conventional issues like collection, preservation and transfer could decrease the quality of valuable samples. This has created a roadblock in exploring clinical samples and samples from extreme environments. This issue has been resolved to a reasonable extent by another third-generation sequencing platform, the Oxford Nanopore MinION™ DNA sequencer [150]. The MinION system incorporates a protein nanopore embedded on an electrically resistant polymer membrane wherein an ionic current is passed through the nanopore by setting a voltage across this membrane **(**Figure 3**C****)**. A characteristic disruption in current when a DNA or RNA strands or single nucleotides are driven through the nanopore allows sequences to be read out in real time resulting in longer read lengths. Importantly, the MinION system is a handheld, portable system that provides the agility to sequence samples from extreme conditions. Many recent studies prove the agile applicability of MinION sequencing, including characterizing of Ebola virus samples in its recent outbreak in West Africa, studying Zika virus in northeast Brazil, or the multilocus sequence typing genotyping of vancomycin-resistant *Enterococci* [63, 151]. At present, MinION provides >10 Gbp yield per flow cell with >10 times longer read coverage of even low abundant genomes (<1%) [151, 152]. Notably, higher error rates (~30%) observed for early MinION systems have been reduced to a moderate range between 2 and 13%. But large-scale applications of MinION are still limited due to higher error rates compared to shotgun sequencing, low coverage and high level of interrun variabilities.

Nonetheless, recent papers have suggested a hybrid approach for MinION metagenomics applications. These studies showed that challenges in metagenomics cannot solely be solved using longer reads but that more accurate reads are required for a better resolution. Recently, hybrid approaches were frequently applied for whole genome assemblies of clown fish and *Saccharomyces cerevisiae* genomes [153, 154]. Moreover, investigations on gut metagenomes of patients undergoing antibiotic treatment and studies on identifying native forms of multiple RNA viruses also utilized a hybrid approach for obtaining microbiome data [155, 156]. Eventually, the emerging sequencing technology and the need for hybrid methodologies have led to the development of BusyBee, a reference-independent binning Web tool that accepts Illumina-assembled contigs and long reads from PacBio and MinION [157].

### Computational best-practice protocol for microbiome acquisition

**Figure 4:**
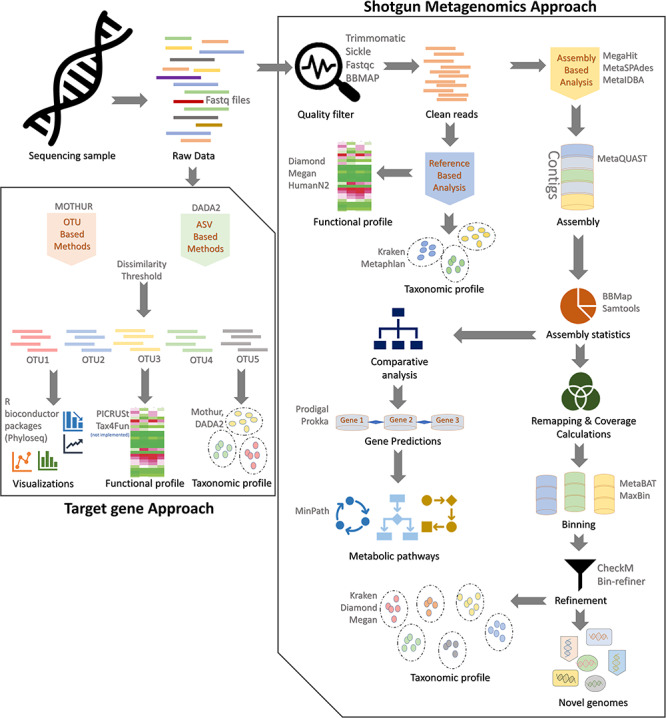
Best-practice protocol for the acquisition and analysis of targeted amplicon and shotgun metagenomics data from sequencing to functional annotation. The basic flow of experimental steps followed by downstream preprocessing and analysis steps is shown. At each step, the optimal tools utilized during the process are shown as well. All scripts are available at https://github.com/grimmlab/MicrobiomeBestPracticeReview.

To simplify the process of conducting such studies, we implemented a best-practice workflow. These standardized protocols will help to obtain more robust and reproducible analyses for target gene and shotgun metagenomic sequencing data. An overview about the individual steps of the workflow is presented in Figure 4.

All scripts and a detailed documentation are available on GitHub under the following link (https://github.com/grimmlab/MicrobiomeBestPracticeReview).

#### Target gene approach

Raw reads are quality filtered and processed by either *OTU-*based (mothur) [159] or *ASV*-based (DADA2) [158] methods utilizing a dissimilarity threshold (left panel, Figure 4) are used for OTU-based data processing and analysis in the section below:

(i) Taxonomy: The OTU table generated from the processed reads is used for profiling microbial abundance present in the sequencing data. Microbial communities are identified through a rigorous protocol that results in multiple pangenome alignments using customized databases such as SILVA, Greengenes and RDP of various genes families.
(ii) Functional annotation: Using the output from mothur or DADA2, the functional profile of identified microbial communities can be predicted using Tax4Fun or PICRUSt [161, 162]. While Tax4Fun is an R-based algorithm utilizing SILVA as reference, PICRUSt is a bioinformatics pipeline that clusters protein sequences based on KEGG or COG gene families and 16S rRNA gene copy numbers. Both provide approximation of functional profiles in a given dataset.
(iii) Data visualization: The resulting OTUs and the dissimilarity indices can be then utilized for assessing differences within and among samples and visualized using R Bioconductor package phyloseq [163].

#### Shotgun metagenomics approach

This approach comprehensively catalogues all genes from a diverse pool of microbial genomes present in a sample. Current sequencing platforms are broadly classified as either short-read (250–300 bp; Illumina) offering higher sequencing depths or long-read (500–4000 bp; PacBio and Oxford Nanopore) technologies offering better contig assembly. Eventually, either of these still relies on optimal sequence processing including proper assembly:

(i) Quality filtering: Metagenomic sequencing harbors large heterogeneity in the reads as compared to the target gene approach. Heterogeneity in metagenomics reads mostly pertains to poor quality or ancillary adapter/hairpin sequences that are removed during the quality filtering steps. For short-read sequencing platforms such as Illumina and Solexa, both paired-end and single-end reads could be optimally trimmed using Trimmomatic, Sickle and BBTools. Apart from dedicated modules for trimming short-read sequences, it can well be utilized for long-read sequences too. The quality-filtered processed reads are further passed through the *assembly-based* and *reference-based analysis* (right panel, Figure 4).
(ii) Reference-based analysis: This analysis mostly involves alignment with databases (NCBI or a customized repository like SILVA) to generate taxonomic catalogue of the communities:
  (a) Taxonomy: Compositional profiling of communities from metagenomic sequencing data can be optimally done by either using unique clade-specific marker genes identified from 3000 reference genomes (MetaPhlAn) or by exact alignments of *k*-mers alongside a classification algorithm (Kraken).
  (b) Functional annotation: The functional profiling of metagenomic communities can be optimally performed using HUMAnN2 or Megan pipelines. HUMAnN2 implements a biphasic alignment screen with MetaPhlAn, followed by functionally annotated pangenomes of the identified species. Megan does annotations using seed classifications through KEGG orthology and COG/NOG classifications. For long reads the DIAMOND sequence aligner can be used alone or with Megan to perform pairwise and frameshift alignments.
(iii) Assembly-based analysis: This is a more comprehensive analysis utilizing *de novo* assemblers for metagenomic sequencing data. The three most optimal assembling algorithms are MegaHit, MetaSPAdes and MetaIDBA described in preceding sections (right panel, Figure 4).
  (a) *Contig assembly*: The assembled reads are clustered into contigs and evaluated by MetaQUAST [164] that compares them with metagenome assemblies based on alignments to close references.
  (b) *Assembly statistics*: This step is a prerequisite of remapping/coverage calculations and comparative analysis. SAM (Sequence Alignment Map) tools optimally perform sorting and indexing alongside alignment generation. Similarly, for very large genomes, BBMap could be preferred that can equally handle both short- and long-read sequences from Illumina, PacBio, or MinION.
  (c) *Comparative analysis*: Comparative analysis incorporates algorithm-based gene predictions and metabolic pathway identifications. Prokka annotates the data by predicting genes using Prodigal and then performs functional annotation on these genes [165]. For homology search Prokka uses CDD, PFAM and TIGRFAM databases on prodigal translated protein output. Further, the MinPath algorithm [166] could be implemented for biological pathway reconstructions based on protein family predictions.
  (d) *Binning*: Following remapping and coverage calculations, binning or grouping of generated contigs is done before further downstream analysis. Either MetaBAT with an adaptive binning algorithm or MaxBin that utilizes an EM algorithm could be used for metagenomic contig binning.
  (e) *Refinement*: Post-binning remapping and refinement steps are utilized for generating taxonomic profiles and annotation of any novel genomes present in samples. Both CheckM [167] and bin-refiner [168] are optimally used for estimating genome completeness and contamination. Taxonomic profiles and novel genome identification can be optimally performed using the above-described Kraken [114] and Diamond algorithms [121] with or without the Megan pipeline [117].

#### Downstream and statistical analysis

Analyzing microbial data is challenging due to its large and multivariate data structure. In general, it is difficult to provide a best-practice pipeline for straightforward statistical analysis because it highly depends on the core objectives of the study and the underlying hypothesis. However, there had been tremendous efforts to develop tools that facilitate these analyses. Two widely used tools for statistical downstream analysis on microbial data are Calypso [169] and MicrobiomeAnalyst [170]. Calypso can be used to perform compositional analysis of large metagenomics datasets with univariate and multivariate statistical tests and data representations. MicrobiomeAnalyst provides various options for community profiling, functional profiling and metabolic network visualization for both amplicon and shotgun metagenomics data. Apart from these, there are additional statistical analysis and visualization tools, including Metaviz and PUMA [171, 172]. In addition, a detailed overview on statistical analysis for microbial data is described in recent reviews and book chapters [173, 174, 175, 176].

## Future challenges

Current computational developments are expected to produce efficient and scalable solutions. However, it is still vital to implement multiple high-throughput strategies to reaffirm the preciseness of genomic findings. To correctly describe genomes with their respective environmental functions, biases in sampling saturation should be addressed by improving the resolution of genomic analysis. This necessarily requires more profound analysis of low-complexity communities through comparatively more modern metatranscriptomics and metaproteomics technologies. This will help to address previously unobtainable biological information from microbiomes that would eventually aid in creating better therapeutic and biotechnological applications. Metatranscriptomics is the analysis of community transcripts isolated directly from multiple environments showing variability in the microbiome compositions. Metatranscriptomics data directly correlate with the taxonomic signature of communities and its function by profiling mRNA transcripts generated under different environmental conditions. To aid high-resolution analysis, higher coverage of genomic information from environmental conditions by shotgun metagenomics could be fused with metatranscriptomics. Metaproteomics on the other hand involves analysis of microbiome-associated protein profiles providing information on the function directly under different environmental conditions. Nevertheless, community protein profiling relies more heavily on the preciseness of metagenomics data. Mass-spectrometric analysis of different peptides generated from an environmental sample could be matched with the predicted proteins from metagenomics analysis. Overall, the future of both target gene and metagenomics projects not just relies on emerging computational resources but also on more in-depth and complementary sequencing methodologies. This will eventually help in reaffirming the reliability of sequencing data and for establishing more comprehensive approaches for delineating the functional profiles of environmental samples.

## Conclusions

Both target gene and metagenomic sequencing approaches are key to decipher a plethora of roles which are played by environmental microorganisms. However, both sequencing and computational methods still suffer from many biases that are due to errors in sample handling, experimental errors and downstream bioinformatics analysis. Thus, improvements in sequencing technologies and the development of new computational tools and algorithms should always be based on prior knowledge, e.g. known caveats at each sample processing step. Factors that potentially influence preprocessing, as well as downstream analysis of both short-read and long-read data including sample preparation, sequencing, binning, assembly and functional annotations, should be catalogued precisely. Herein, we have attempted to list challenges and best-practice protocols utilized during microbiome acquisition using 16S rRNA and metagenomic sequencing. This is important due to the large and expanding paradigms of computational tools that have been developed in recent years for analyzing long- and short-read sequencing data. Here, we provide a workflow of optimally tested tools available for processing sequencing samples, estimating microbial abundances, and classification, assembly and functional annotations. In addition, we also discussed the experimental challenges with a systematic review of steps involved in 16S rRNA and shotgun metagenomics. The experimental challenges mainly account for factors responsible for contamination in isolated microbial genomes and resulting variations in microbial profiles. Although gradual improvisation of these factors has been implemented, extensive and multilayered, sequencing data remain prone to errors at various levels. Hence, we believe that utilization and awareness of integrated methods described here will not just help to improve the reliability of sequencing outcomes but would also reduce variability in the data generation and processing steps.

## Supplementary Material

SupplementaryData_bbz155

## Notes

Richa Bharti is a postdoctoral researcher at TUM Campus Straubing for Biotechnology and Sustainability and Weihenstephan-Triesdorf University of Applied Sciences. Her research is focused on analyzing metagenomics and RNA-Seq data and developing bioinformatics tools and pipelines for microbial data.

Dominik Grimm is a professor for bioinformatics at TUM Campus Straubing for Biotechnology and Sustainability and Weihenstephan-Triesdorf University of Applied Sciences. His research is focused on developing bioinformatics and machine learning tools for analyzing bio(medical) data.

## Notes

Key Points

- NGS has revolutionized scalability, speed and cost-effectiveness to perform a wide range of studies, including the analysis of microbial communities associated with host and environment.
- For studying microbial composition, target gene and metagenomic sequencing are commonly used methods to investigate the composition of both genes and novel genomes across multiple environments.
- Experimental setup and data analysis are error-prone tasks, which might lead to high variability in short- and long-read sequencing and data analysis.
- Standardizing sequencing data acquisition and data analysis is crucial for obtaining comparable and reproducible results. This article provides an overview about experimental and computational challenges, including best-practice protocols for amplicon and metagenomics data analysis.
