# Supplementary material for: Current challenges and best-practice protocols for microbiome analysis
Source: Brief Bioinform. 2019 Dec 18;22(1):178–93. doi: 10.1093/bib/bbz155 (PMC7820839; doi:10.1093/bib/bbz155)
Supplement: SupplementaryData_bbz155 [file supplementarydata_bbz155.docx]

**Supplementary Table S1: Sample Metadata file**

| **1. Sample ID - Sequencing Facility** | |
| --- | --- |
| Sequencing ID | RB7486 |
| Field Name | RB7486/TUM Campus Straubing |
| Description | Sample submitted by the TUM Campus Straubing, Germany |
| Privacy Risks | No |
| GenBank Structured Comment Synonym | GYA* |
| Data Categories | Sample shipment |
| Syntax | Alphanumeric |
| Data Source | HPIND |
| Comments | This data would be embedded in the GenBank record as a dbxref for linkage of HSC metadata records with GenBank sequences |
| **2. Nucleic Acid Extraction Method** | |
| Sequencing Assay Field ID | GA4 |
| Field Name | Nucleic Acid Extraction Method |
| Description | Experimental protocol used to isolate nucleic acid fraction from the submitted sample for sequencing reaction. |
| Privacy Risks | No |
| Data Categories | Sequencing Sample Preparation |
| Example Values | Illumina standard method; CTAB/chloroform |
| Comments | Three out of seven samples were dry and were not used. |
| **3. Nucleic Acid Preparation Method** | |
| Sequencing Assay Field ID | GA5 |
| Field Name | Nucleic Acid Preparation Method |
| Description | Details of preparation and amplification of DNA samples for sequencing including any other relevant molecular biology protocols done prior to sequencing. |
| Privacy Risks | No |
| Other Synonyms | Nucleic acid preparation by GSC; including amplification procedure |
| Data Categories | Sequencing Sample Preparation |
| Example Values | Standard 454 LC |
| Comments | Not applicable |
| **4. Sequencing Method** | |
| Sequencing Assay Field ID | GA6 |
| Field Name | Sequencing Method |
| Description | Experimental protocol for deriving sequence data from samples. Type of sequencing used is Illumina. |
| Privacy Risks | No |
| Other Synonyms | Sequencing method (Illumina SBS) |
| Data Categories | Sequencing Assay |
| Data Source | HPIND |
| Comments | Not applicable |
| **5. Assembly Name** | |
| Sequencing Assay Field ID | GA7 |
| Field Name | Assembly Name |
| Description | A unique name given to a specific assembled genome build. |
| Privacy Risks | No |
| Other Synonyms |  |
| Data Categories | Data Transformation |
| Data Source |  |
| Comments | Genbank ID requested |
| **6. Assembly Method** | |
| Sequencing Assay Field ID | GA8 |
| Field Name | Assembly Method |
| Description | Software or pipeline used to assemble individual sequence reads into larger contigs. |
| Privacy Risks | No |
| Other Synonyms | Assembly (Assembly method, estimated error rate and method of calculation) |
| Data Categories | Data Transformation |
| Data Source |  |
| Comments | N.A. |
| **7. Depth of Coverage - Average** |  |
| Sequencing Assay Field ID | GA9 |
| Field Name | Average depth of Coverage |
| Description | Depth of sequence coverage based both on external and internal measures of genome size. |
| Privacy Risks | No |
| Other Synonyms | Average depth of sequence coverage |
| Data Categories | Data Transformation |
| Data Source |  |
| Comments | Value, min, max |
| **8. Annotation Algorithm** | |
| Sequencing Assay Field ID | GA10 |
| Field Name | Annotation Algorithm |
| Description | Computational algorithm for identifying sequence features in the assembled contig sequence |
| Privacy Risks | No |
| Other Synonyms | Annotation source |
| Data Categories | Data Transformation |
| Data Source |  |
| Comments | Temporarily stored until submission |
| **9. Annotation Pipeline** | |
| Sequencing Assay Field ID | GA11 |
| Field Name | Annotation pipeline |
| Description |  |
| Privacy Risks | No |
| Other Synonyms |  |
| Data Categories | Data Transformation |
| Data Source |  |
| Comments | Genbank id requested |
| **10. GenBank Record ID** | |
| Sequencing Assay Field ID | GA12 |
| Field Name | GenBank Record ID |
| Description | Unique identifier of the submitted GenBank sequence record(s). |
| Privacy Risks | No |
| Other Synonyms |  |
| Data Categories | Data Transformation |
| Data Source | HPIND |
| Comments | Not available until full sequence submitted |
